# Conversations with JCI editors in chief

**DOI:** 10.1172/JCI187017

**Published:** 2024-10-15

**Authors:** Ushma S. Neill

On the occasion of the 100th birthday of the *JCI*, the living editors in chief of the *Journal of Clinical Investigation* (Table 1) were asked to reflect on their time as editors and their favorite memories. The full video is available on the *JCI* website at https://www.jci.org/videos/cgms, and the unedited entirety of the editors answering each question is available on the *JCI* YouTube channel at https://www.youtube.com/@jclininvest.

*JCI*: How did you come to be the JCI editor in chief (EiC)?

Stuart Kornfeld: It was a fluke. Phil Majerus, who was the EiC, was elected to be president of the ASCI. He stepped down and I was the closest person to him. I volunteered to do it while he was doing presidency at ASCI. It turned out it was a lot of fun. A lot of work, but a lot of fun.

Joseph Avruch: I had a great time serving as a *JCI* associate and interim editor, primarily because the weekly meetings were actually fun, in comparison to the mostly grim, boring, or simply deadly nature of nearly all other administrative conclaves. Tom Stossel set the tone, which was informal and nonstuffy, but scientifically rigorous and entirely respectful of the manuscripts under review. The *JCI* then had a reputation for publishing high-quality, well-defended biochemical/physiological research that was, broadly speaking, clinically relevant; however, the *JCI* did not attract work that authors considered “hot” or really novel. Nevertheless, the discussion among the associate editors of the papers, the reviews, and the relevant field were lively and wide ranging and greatly amplified the interest and value of the meetings for the editors themselves.

Bruce Scharschmidt: I didn’t apply; I didn’t volunteer. I got a phone call in 1985 from Dr. Joe Goldstein, who was then president of the ASCI. Later that year became a Nobel Prize winner. He asked me if I was willing to serve as editor. I have no idea what the selection process involved, perhaps a council of elders followed by a puff of white smoke.

Ajit Varki: It was a most unexpected honor and at a relatively early stage of my career. As I understand it, my selection as editor was the first time there was an advertised opening in a national competition. Many institutions responded with letters of intent, but since the existing editorial office was at UCSF, no other UC schools were intending to apply. But the leaders at UCSD suggested that I should apply just to make us look good. I initially demurred but agreed when I was assured privately that I would not have any chance since I was a foreigner.

Paul Insel: When UCSD was considering applying, I volunteered Ajit, as I knew he would be perfect for this. After he was picked, he said, “You are the one that got me into this, so you will be one of the associate editors.” About three or four years in, Ajit was asked to be the acting director of the cancer center, so I had to serve as EiC.

Stephen Weiss: I had gotten calls from the chairman of medicine’s office encouraging me to apply and the second time the chair — Tachi Yamada — said, “Have you rethought about entering the lottery to see if you could become the next *JCI* editor and bring it to Michigan?” And very quickly, I said, “No, I thought about this before, but I think this is going to be so disruptive to my academic career that this is probably not the best choice for me.” There was a pause, and they said (it was one of Yamada’s representatives), “No, no, you don’t understand, Steve. Dr. Yamada is not asking you if you’re interested in doing this. He is telling you, you have to do it.”

Andrew Marks: I can’t really remember exactly what drove me to do this, but there was a family connection. My dad [Paul Marks] had been the *JCI* editor, so I was very familiar with the journal, and it felt like it was a good time to bring it back to Columbia. And I think most exciting to me was the idea of working closely with a really great group of colleagues, getting to know them better and sharing science with them. I think those were the motivating factors. Of course, I had no idea what I was getting myself into.

Laurence Turka: The call for editorships came out in the fall of 2005, and at that point, I had been at Penn for 11 years, so I knew people pretty well and I was fairly well connected. I had also been the renal division chief for seven years; I don’t like to do things for too long. I was already thinking, what am I going to do next? I thought this editorship could really be a lot of fun together with great colleagues at Penn.

Howard Rockman: A number of my colleagues noticed the request for application, and we got together and they encouraged the application from Duke. And they thought at the time that I would be a strong candidate; we could put together a very strong board. Almost everyone that we approached was really excited to do it.

Gordon Tomaselli: Hopkins was really quite keen on hosting the *JCI*. We’d never hosted it before, and I was asked by then Dean Paul Rothman to assemble a team and put together an application. I was a little bit reluctant. My area of research wasn’t one that was highly featured in the *JCI*. In fact, at the time, I think I’d published one paper in the *JCI*, although submitted a number more. And moreover, of the prior four editors, three of them were researchers in cardiovascular disease. So I thought this wasn’t really gonna work out, but it did.

Rexford Ahima: Happenstance. I had spent many years at Penn and had served as an associate editor under Larry Turka. In 2016, I came to Hopkins to lead the endocrine division and serve as a Bloomberg Distinguished Professor. In 2017, Gordon Tomaselli became the EiC and asked me to serve as his deputy editor together with Arturo Casadevall. Things went well for a year, and then Gordon decided to step down and move to Einstein. I decided to step up, and we had to go through an interview process with the ASCI board.

Elizabeth McNally: I had a longstanding interest in the *JCI*. I was a former president of the ASCI and as a long-time author in the *JCI*, I have always sent what was our best work to the *JCI*. It is a journal that has meant a lot to me because it’s where I like to publish things. It’s where I like to read things. When I first applied, I was at the time moving from University of Chicago to Northwestern and it was one of the things I pitched to the dean at the time saying, “If I come, I really want to apply to be EiC and I hope I have your support to do it.”

*JCI*: What was the publishing landscape like when you took over as EiC?

Kornfeld: It’s amazing how it’s changed. Each subspecialty had its own journal, but the *JCI* had the reputation of being above all those and was competitive at that time, I think, even with *Science* and *Nature*, which were considered the top general journals.

Scharschmidt: Open access was just being contemplated; we didn’t have today’s myriad of journals. The tectonic plates of publishing were starting to move, and we were about a decade into the new era of molecular genetics and medicine. The competition was becoming increasingly fierce and crowded for that new work: Cell Press had just been spun off. *Science* and *Nature* were going after that work. *Nature Genetic*s was born during the time of our tenure.

Insel: At that time, the *JCI* was the king of translational research. During the UCSD days, *Nature Medicine* was launched and they were a major challenge for the *JCI*.

Weiss: *Nature* had launched already *Nature Medicine* and *Nature Genetics*. So we already knew there was almost too much competition. So we thought hard about how to increase the profile and visibility of the journal.

Marks: *JCI* was one of the first journals to really venture into open access and to champion that. It was the biggest change that occurred around that time in scientific publishing and was obviously very important.

Turka: In 2007, open access was hotly debated. We were open access, but people had to pay to help support the journal. That was a model that made the *JCI* budget livable.

Impact factor was a huge issue. We were getting top-tier papers, but I was never under the illusion that we were always everyone’s first-choice journal, but we were many people’s first-choice journal. I was always very proud of what we published, but what engendered a lot of discussion among the editorial board is what’s the purpose of the *JCI*. Is it to have a high impact factor, or is it to publish as many good science papers as we can?

Rockman: It was an interesting time. We started our tenure in 2012, and it was my opinion at the time that many editors of journals, of some journals, felt that it was their job to operate as gatekeepers, as if the science and the scientific process needed protection from bad actors. It was a time where issues of reproducibility had surfaced, and there was also the rise of the sleuths scouring the literature for potential malfeasance. As editor, I had a different view. I set out a culture on the board where we’d publish science that was rigorous and advanced knowledge, but we aimed to limit excessive experimentation.

Tomaselli: A year before we started, Howard Rockman started *JCI Insight*. He wound up manning that with a team from Duke for about three and a half years, well into our term, before he turned it over to Kathy Collins at the University of Michigan. So the biomedical landscape was continuing to evolve. There weren’t as many so-called daughter journals as there are now, but they were increasing in number, seemed like every week there was a new daughter journal that was popping up. Towards the end of our term, *JCI* became fully open access. The other thing that was happening was preprint repositories like bioRxiv were maturing. MedRxiv in 2019 became kind of established.

*JCI*: Do you have any favorite personal memories of your time as EiC?

Kornfeld: What I remember is the camaraderie. We all realized we were in for a lot of work, but we were happy to do it. We all felt that we learned a lot about medicine and how to do research and various techniques, which we would not have gotten without being on this board.

Scharschmidt: We had some staff perfectly tailored to that job. There was a young Irish woman with a charming lilt in her voice. She was the one who called to solicit the reviewers. No one could say no to her. Everybody said, sure, I’ll review that paper, but when it was time to get the review back and we didn’t have it, we had another guy in the office, actually a charming fellow, really warm heart, but he’d been an ex-Israeli military pilot. And he could be pretty fearsome on the phone. And those reviewers listened and they responded. So as a result, we got fewer excuses for late reviews, but some were real doozies and they were so good that I actually read them aloud at the annual meeting when I was giving my report: “I took the manuscript to a meeting in Europe, and I couldn’t find a typewriter.” “I lost the manuscript on the New York state thruway.” “I lost the manuscript in the men’s room.” “I lost the manuscript in the Pleistocene layer on my desk.”

Varki: My favorite memory is that of introducing the *JCI* free online in the early days of the internet. Prior editors such as Phil Majerus and Bruce Scharschmidt had put away invested funds in a rainy-day account. I continued this account and luckily got us out of the market just before the dot-com crash. This fund approached enough that I could release the *JCI* on the web as free access to everyone. I didn’t realize that I had actually created something which would later many years be called open access. People have forgotten that *JCI* did it first.

Insel: We developed a shorthand, which was, “NFU” or “not for us,” and we would immediately be able to reject out of hand, NFU. There was also a story that Wash U EiC Phil Majerus had handled a paper wherein there had been a split decision and so he secretly became reviewer three and criticized the paper considerably. The authors came back saying they could address the concerns of reviewers one and two, but reviewer three was an idiot and didn’t know what he was talking about. Majerus responded with some version of “F you!” We used to call that the “Phil Majerus response” that we would sometimes say in our meetings but we never said that to any author.

Marks: The biggest impact for me personally was probably my famous Big Lebowski editorial. When I look back on that I think it did serve a purpose by energizing thought around how we can make the NIH more responsive to the needs of the scientific community, which was my goal.

Turka: I do remember a couple of specific comments that came with reviews: some irate author wrote back when a paper had been rejected and said, “If I sent in a paper that I had a dog that talked, you would come back and say, yes, but what’s the mechanism?” I do remember a faux pas that I committed wherein I accidentally cc’d somebody on an email where I was complaining that I was getting sick and tired of their whining, and they should get a life. Oops!

Ahima: COVID was a challenge, and we had to figure out how to carry on with our business. Many members of our editorial group were ID specialists or public health specialists who had other things to do. Our submission rate also increased about 66% during that time.

My other favorite memory has to do with Gregg Semenza being awarded a Nobel Prize in Physiology or Medicine in 2019. I don’t think it’s ever happened for most journals to have a senior member receive such an award. The announcement came on Monday, October 7th, 2019, and we had a weekly meeting on a Tuesday. Gregg didn’t show up because he had to deal with the press. But the following week, he came to the editorial board meeting. It shows you how dedicated he was to the journal.

*JCI*: Do you have any favorite research memory of your time as editor?

Scharschmidt: There was one a real head spinner and not for scientific reasons. The paper was from a group in Paris. It was really groundbreaking; we loved it; we accepted it. It was on its way to publication when we got a letter also from Paris implying that the work had been fabricated. We paused publication while looking into things, and it turned out that the manuscript was the innocent bystander of a love triangle and the letter claiming fraud was from the jilted paramour of one of the authors.

Varki: My top choice would be the papers by Mike Brown and Joe Goldstein who won the Nobel Prize in Medicine in 1985. They are long-term ASCI members, and they are ardent supporters of the *JCI* and the ASCI. I particularly remember a 1993 paper that they published while I was editor on hypercholesterolemia in LDL receptor knockout mice and its reversal by adenovirus-mediated gene delivery. This followed another in 1994 in *JCI*, the massive xanthomatosis induced by cholesterol feeding these mice.

Insel: There was a paper about grapefruit juice and its impact on drug absorption and degradation. We published it, but the amount of grapefruit juice that you’d have to drink to have an impact is tantamount to eight ounces two to three times a day. But this was very interesting pharmaco-biolog.

Ahima: Unsurprisingly, it’s COVID related biology. At the time the epidemic was really at the peak (April 2020), we had a paper from a group of scientists from China describing the clinical and immunological features of this new syndrome. And as you can imagine, it was not typical *JCI* because at that point, there were no mechanistic insights.

We had a very heated discussion and to their credit, this group had some pieces in place, especially the immunology, which competitors did not have. It turned out to be one of the defining papers in the field. It’s very much referenced. I think they are up to close to 3,000 citations since 2020.

*JCI*: What do you think has been the impact of the *JCI* on the broad biomedical community?

Kornfeld: Well, I think it served as, or it serves still, as an example of high-quality, carefully done biomedical research, asking problems, studying problems related to human disease and physiology. That was how we felt about it back in 1981. I don’t think that’s really changed.

Scharschmidt: The *JCI* from the start is focused on translational science work in the space ranging from discovery to early demonstration of benefit in humans. It’s raised the visibility of that whole era. And of course that plays to the strength of physician-scientists, which is what the ASCI is all about.

Marks: What stands out with the *JCI*, is that it’s a journal edited by scientists for scientists, and I think has a great brand name for many, many generations of scientists, not only physician-scientists, but basic science as well. One of the great strengths of *JCI* is that it changes editorial boards every 5 years. You get a new look, but it doesn’t change the journal fundamentally. It’s had a great tradition to it, and that bodes well for the next 100 years.

Ahima: My answer here will obviously be biased. When it comes to clinical investigation, the *JCI* is the gold standard. I can’t think of any other journal that comes close.

## Figures and Tables

**Table 1:**
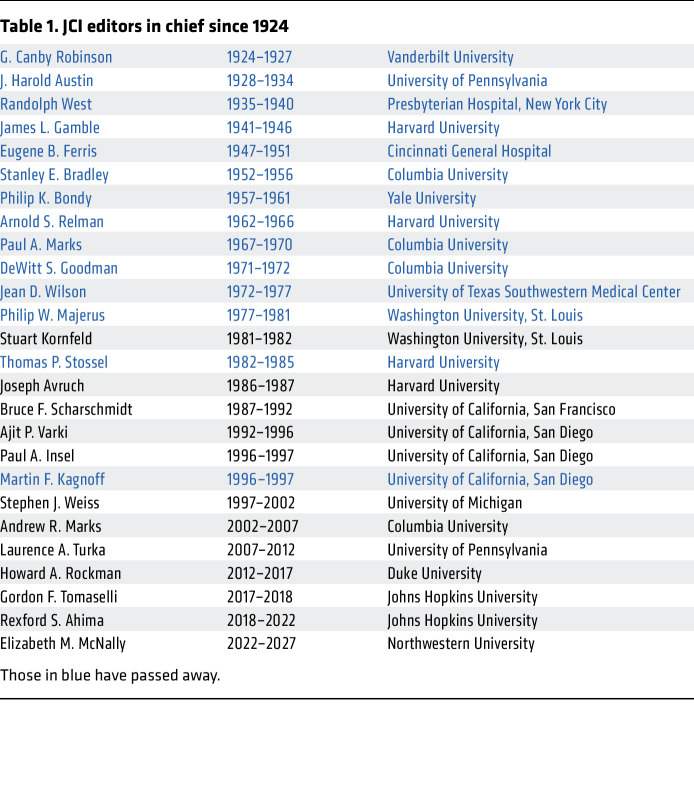
JCI editors in chief since 1924

## Version 1 10/15/2024

Electronic publication

